# Parental perspectives of episodic irritability in an ultra-rare genetic disorder associated with *NACC1*

**DOI:** 10.1186/s13023-023-02891-3

**Published:** 2023-09-04

**Authors:** Kelly Schoch, Allyn McConkie-Rosell, Nicole Walley, Vikas Bhambhani, Timothy Feyma, Undiagnosed Diseases Network, Carolyn E. Pizoli, Edward C. Smith, Queenie K.-G. Tan, Vandana Shashi

**Affiliations:** 1 Division of Medical Genetics, Department of Pediatrics, Duke University School of Medicine, Durham, NC USA; 2 Division of Genetics and Genomic Medicine, Children’s Hospital and Clinics of Minnesota, Minneapolis, MN USA; 3 Gillette Children’s Specialty Healthcare, Saint Paul, MN USA; 4 Division of Neurology, Department of Pediatrics, Duke University School of Medicine, Durham, NC 27710 USA

**Keywords:** NACC1, Extreme irritability, NECFM, Episodic irritability, Agitation, Paroxysmal sympathetic hyperactivity, Neuroirritability

## Abstract

**Background:**

A recurrent de novo variant (c.892C>T) in *NACC1* causes a neurodevelopmental disorder with epilepsy, cataracts, feeding difficulties, and delayed brain myelination (NECFM). An unusual and consistently reported feature is episodic extreme irritability and inconsolability. We now characterize these episodes, their impact on the family, and ascertain treatments that may be effective. Parents of 14 affected individuals provided narratives describing the irritability episodes, including triggers, behavioral and physiological changes, and treatments. Simultaneously, parents of 15 children completed the Non-communicating Children’s Pain Checklist-Revised (NCCPC-R), a measure to assess pain in non-verbal children.

**Results:**

The episodes of extreme irritability include a prodromal, peak, and resolving phase, with normal periods in between. The children were rated to have extreme pain-related behaviors on the NCCPC-R scale, although it is unknown whether the physiologic changes described by parents are caused by pain. Attempted treatments included various classes of medications, with psychotropic and sedative medications being most effective (7/15). Nearly all families (13/14) describe how the episodes have a profound impact on their lives.

**Conclusions:**

NECFM caused by the recurrent variant c.892C>T is associated with a universal feature of incapacitating episodic irritability of unclear etiology. Further understanding of the pathophysiology can lead to more effective therapeutic strategies.

**Supplementary Information:**

The online version contains supplementary material available at 10.1186/s13023-023-02891-3.

## Background

The nucleus accumbens associated 1 gene (*NACC1* [MIM: 610672]) is a member of the BTB/POZ domain-containing gene family and encodes NAC1, a protein that functions as a transcriptional regulator. Our group, as part of the Undiagnosed Diseases Network (UDN), previously reported a recurrent de novo missense variant in *NACC1* (NM_052876.3:c.892C>T, p.(Arg298Trp)) in seven children, resulting in severe to profound intellectual or developmental disability (7/7), epilepsy (7/7), bilateral congenital cataracts (5/7), postnatal microcephaly (5/7), feeding difficulties and/or feeding intolerance (7/7), stereotypic movements (6/7), sleep disorder (5/7), delayed myelination (4/7), and cyclical bouts of irritability (7/7) [MIM# 617393; Neurodevelopmental disorder with epilepsy, cataracts, feeding difficulties, and delayed brain myelination (NECFM)] [1]. Although not included as a key feature in the acronym NECFM, recurring episodes of excessive crying, tactile aversion, breath-holding spells, and inconsolability had been reported. Since this publication, more individuals have been diagnosed with NECFM, and it is evident from communications from parents and providers, that the periodic irritability is ubiquitous in individuals with the recurrent c.892C>T variant, manifests early in infancy, is debilitating and is thus a source of extreme hardship for families. The episodes are challenging to treat.

The term “irritability” was chosen to characterize these behaviors in this study because it is defined as an abnormal responsiveness to a broad variety of stimuli, which could include pain, fright, drug, emotional trigger, or medical condition [2]. An alternative term that would be appropriate is “neuroirritability” which has been used to describe persistent or recurrent episodes of pain arising in the CNS, once nocioceptive sources of pain have been ruled out [2]. However, since we are not sure if the cyclical episodes are solely pain-related, we use the broader term of “irritability” to describe these. Severe irritability has been observed in many neurodevelopmental and neurologic disorders [2–7], although not typically episodic as in NECFM. These include a few rare genetic disorders, such as Krabbe [8], Juvenile Neuronal Ceroid Lipofuscinosis (JNCL) [9], and Rett syndrome [10, 11]. In Krabbe disease, the etiology of irritability is uncertain, but may be pain-related due to peripheral neuropathy [8]. Children with Baker-Gordon syndrome, an ultra-rare disorder caused by *SYT1* variants, experience agitated phases, which are unprovoked and may include screaming, increased involuntary movements, chest-beating, and minor self-injury including chewing on fingers and hands [12]. The length of the episodes varies from minutes to days, but unlike NECFM they were not described during infancy [12]. The episodic irritability observed in NECFM appears to be unique in that it is cyclical, extreme, seemingly unprovoked, manifesting in infancy, and long-standing.

Since the cause of the irritability is unknown in NECFM and children are unable to communicate, we considered possible sources, beginning with pain. Pain is defined as an unpleasant sensory and emotional experience associated with actual or potential tissue damage [2]. In children with severe neurological impairments, there are many different causes of pain, such as nocioceptive (arising from damage to non-neural tissue), or neuropathic (arising from disruption of the somatosensory nervous system, such as central neuropathic pain and visceral hyperalgesia) as well as autonomic dysfunction [2] and we sought to obtain more information on pain through a validated pain survey. However, the possibility remains that the cyclical episodes in NECFM are caused by an underlying etiology other than pain. Due to the universal, unique, severe and incapacitating nature of the periodic irritability and the tremendous medical impact on the affected individuals and psychological impact on the families, we designed a study to describe the episodes in further detail, understand the clinical correlates and determine if there were interventions that were effective in mitigating these, from the perspectives of parents caring for these individuals.

## Results

Nineteen parents of 15 individuals with the recurrent *NACC1* variant c.892C>T participated in this study. All families (15/15) completed the NCCPC-R, and 14/15 families completed the parental narratives. Participants resided on four continents (North America, Europe, Asia and Australia) and their details are provided in Table 1.

**Table 1:** Demographic information for respondents and the affected child

Demographic variables	Respondent (N = 19)	Affected child (N = 15)
Gender	10/15 mother only (67%) 1/15 father only (7%) 4/15 both parents (27%)	4/15 female (27%) 11/15 male (73%)^a^
Average age (years)	34.0 ± 10.1 (34–72 years)	9.0 ± 6.4 (1–25 years)
Local origin	14 Caucasian, 3 Asian, 2 preferred not to respond	12 Caucasian, 2 Asian, 1 > 1 race
Ethnicity	1/19 Hispanic (5.2%)	1/15 Hispanic (7%)
Age at diagnosis	n/a	Mean: 6.2 ± 6.2 (birth to 25 years)

^a^The skewed female/male ratio is noted, but the gender difference is not as striking in a larger cohort of individuals known to the authors

### Narrative description of irritability episodes

Fourteen families completed the narrative prompts. Almost all (13/14) of the families described episodes of extreme irritability, agitation and inconsolability. One family described their 1 year old child as having had only had one crying episode; however within the next month this family reported that their child was experiencing the typical episodic crying and inconsolability. However, for consistency of the methods, the original narrative provided by the family was used for analysis.

Analysis of the parental descriptions of the irritability episodes demonstrated that they consisted of sequential Prodromal, Peak, and Resolving phases (Table 2). In between these episodes the parents described their children as engaged, happy and content in the setting of significant developmental disability.

**Table 2:** Descriptions and representative quotes from parents characterizing the phases of the irritability episodes

Description of episodes	Representative quotes from parent narratives
*Prodromal phase*: Occurs 1–2 days prior to the peak of the irritability episode Families describe their children as having behavioral or physical changes indicating the impending onset of an irritability episode (13/14). They describe increase in vocalization (7/14), movements (6/14) and stiffness/spasticity (6/14); development of agitation (6/14), jaw clenching (5/14), inappropriate smiles/laughter (7/14), clammy hands and feet (7/14), an unusual smell (3/14), and decreased sleep (6/14)	There are several behavioral and movement symptoms that precede his bad episodes or storms as we call them. We start to notice 1–2 days prior to the cycle these symptoms: sleep disturbances, trouble sleeping, red clammy hands/feet, grinding his teeth, slight increased spasticity, and more sensitivity to his environment such as him giving us warning yells or the inappropriate laughter. The sensitivity to his environment includes not being able to tolerate people talking to him and responding with an inappropriate crazy laughter that actually then turns into a pout and a cry. It's his way of saying, "this is too much loud and too much input and I can't take it anymore, stop." **Family 1** We can understand the bad cycle upcoming when we see a different gaze in the eyes of [our son]. He becomes disoriented, starts to contract muscles, puts his hands in his mouth, increases the salivation, his hands and feet have a cold sweat. **Family 8** Stiffness in limbs, involuntary movement of arms and legs (dystonia) vomiting and nausea and retching, sleeplessness, not wanting to eat or drink. Manic laughing, restless and not sleeping. **Family 5** I notice clammy hands, hot/sweating forehead, more rigidity in his arms and legs, constipation, serious/no smiles. **Family 9** For a while she had a smell, coming from her breath/head, that we'd call the bad day smell. She'd get it the day before the bad days. It was a weird acidic sort of smell. **Family 11** She starts with her right ear getting red and blotches of red on arms, chest, and face which usually signals one coming. She get very stiff and yells, cries, or laughs uncontrollably. **Family 12**
*Peak phase*: The peak of the irritability episode lasts from 1 to 5 days, with the longest reported at 10 days Parents describe the escalation of the episodes in various terms, including “storms”, “bad cycles”, “hypercycle”, “panic attacks”, “spells”, and “bad days”.^a^ The child’s vocalizations become screams/yells (14/14), abnormal movements increase including rolling, thrashing, and posturing (11/14), with stiffness and spasticity (11/14). Signs of agitation (8/14) and gastric symptoms (6/14) increase with some children not being able to eat or drink (5/14). Parents note that their child is unfocused and disengaged (5/14)	It is a painful scream and sometimes more fierce like a neurological crazy cry, like something is driving him crazy/mad and he cannot stop it. **Family 1** When the bad cycle became worst, we lose him, he seems to be absent, and seems to be concentrated only on his pain. He is in another dimension, and we can do very little to calm him, to resolve the situation. **Family 8** Behaviors that emerge or are more severe during an episode—biting or chewing of himself, temperature disregulation (he gets hot very quickly), GI discomfort. He goes from using communication techniques (nods or raising eyebrows) to not communicating at all (except to scream) **Family 4** Irritability getting worse over time, will not sleep without sedative medications, thrashing hands, stomping feet, arching back, lifts head and swing back violently, wriggling around, restless body, turning head side to side, rubbing face -causing skin irritation, stiff, wants to be flat—does not want to sit. Eyes not focusing on anything in particular. Increase work of breathing, stridor is worse. Increased heart rate. Doesn't smile or laugh. Doesn't interact with family/carers. Sometimes bites fingers. Grinding teeth. Dry cracked lips. Head becomes very sweaty. Body temperature increases. **Family 10** During these episodes, she is very irritated, it is better to leave her alone in the dark without noise. She can't stand being touched, talked to, cuddled… No communication is possible. **Family 15** During a hypercycle (irritable cycle), he has involuntary movements and can roll in his bed for hours on end. There is a lack of sleep and that sometimes that lasts about 2–3 days maximum. Exercise (therapy), loud noises, chaos and light will only intensify his involuntary movements. He screams and makes a lot of loud noises. This escalates over 2–3 days till it reaches a peak and then it ends with a sleeping cycle. **Family 7** We have noticed temp changes and he will be diaphoretic, clammy and have low grade fevers that are resolved once the cycle has stopped. **Family 1**
*Resolving phase*: The episodes often end with the onset of sleep, tiredness, and lethargy, typically lasting 1–2 days	In the following days after the bad part of the cycle, [he] sleeps a lot, he is in general sloppy and with a very low tone, he eats and drinks with no rush…. we say that he is recovering from his marathon, from his endurance race. **Family 8** The cyclic bad days would last 3–4 days often culminating in an evening of extreme nausea (nonstop gagging and retching) and then we knew the bad days were ending. She'd sleep that night and wake up better the next day. Often she'd sleep a day or two straight to recover from the bad days. **Family 11** Since she doesn't sleep much during an episode, when she calms down she sleeps a lot for a couple of days. **Family 12**

^a^Parents did not use specific terms to describe the prodromal and resolving phases

### Duration of episodes and change over time

The duration and intervals between episodes varied between the individuals. The parents reported the onset of irritability and inconsolability occurred between birth and 12 months of age. Of the six families who reported onset at birth, three described the inconsolable crying as nearly constant initially, but over time becoming more episodic in nature. The parents described the current frequency of episodes as one to four times per month, and the duration of the episode from hours to days, with the maximum length reported at 10 days. Most of the parents also described a change in the pattern over time, with half noting overall improvement with age (7/14).

Improvements in the episodes were attributed to intervening earlier as parents identified signals that an episode was starting, and reducing environmental triggers (if known) and GI discomfort. Of the families who have children over 3 years of age, none reported that the episodes had worsened since that age, and six reported that the severity of the episodes has remained the same since that time. However, 3 of the 5 families with post-pubertal children commented that the episodes were temporarily worse during puberty.

In the very beginning we couldn’t recognize the start and stop of the single cycle... Then we started to recognize the phases, looking like a sinusoidal curve.... **Family 8**

They are better now, due to a wider array of medications that we use to treat his discomfort and pain (benzos, nausea/GI meds, spasticity, sleep, CBD oil), and also a general approach to treat aggressively sooner rather than later. **Family 4**

### Symptomatic associations, triggers, or external/environmental factors precipitating or preceding an episode

The majority of parents (9/14) specifically stated that they have not been able to identify a consistent trigger. Although a causal link is not certain, five of the families have identified circumstances that are sometimes associated with the the onset of episodes, including GI discomfort, overstimulation, and lack of sleep. However none of these are consistent triggers, and the episodes may occur in the absence of them.

We had done a follow-up over several weeks in 2015 to identify cycles, with her meals, her bowel movements, her crying, her crises, rests, but nothing came out. **Family 15**

He has had many clinic and ER visits earlier on in his life (first 2 years)… looking into GERD, ear infections, gall stones, kidney stones, bladder infections, broken bones, headaches, abdominal distress all to no avail. He has been tested for metabolic or electrolyte changes…. spasticity pain, neuropathic pain, sickness, full moon cycles, weather patterns, tooth pain, intolerance to tube feedings…, but have never been able to link any of these items to be what has caused or triggered an episode. **Family 1**

Eleven of the families reported that their child has had seizures, and of these the majority were under control (9/11). One family was uncertain about whether their child had seizures. Seizure activity was not reported to be consistently related to the irritability episodes.

Some (3/14) feel that there is connection with GI symptoms (constipation, GERD), although it is unclear whether there is a causal relationship or an observed association.

Nothing related to the environment, no hard trigger, no exact association, but in our opinion and experience, just “soft” wrong things that start the bad cycle (examples: a meal not perfectly digestible, eating too fast, too much vegetables, change in habits or about daily routine, strong noises or excess of excitement). **Family 8**

### Intervention during episodes and outcomes

The parents reported limited and variable success in treating the irritabilty episodes with medication, primarily focusing on symptom management. One of the families reported that aggressive bowel cleanout and medications, in combination with early recognition of signs, has reduced the severity of the episodes.

The only medications we've found helpful are those that treat the symptoms... Nothing we have ever tried has prevented or lessened the length of the episodes themselves. They seem to change and evolve over time without any rhyme or reason as to why. **Family 11**

Daily CBD oil (seems to have) helped make the irritability cycles less frequent and not as severe. Maybe. Our initial go-to during cycles is Ativan, and if that does not help him calm down, we try Tizanidine (muscle relaxant). If that doesn't help, we try Clonidine (lowers BP)… Most irritability incidents are resolved with the Ativan. **Family 4**

All 15 families responded to the questions about medication efficacy; they had tried an average of 8.5 ± 12.7 medications (1–53), with a median of 5. However, not all families documented or provided all the medications tried across the child’s lifespan, so this is likely an underestimate. The most common categories of medications tried were psychotropic medications and sedatives (10/15) and anti-epileptic/neuralgic/dystonia medications (11/15), followed by medications for GERD/GI problems (9/15) and anti-adrenergic medications (7/15) (Table 3). Some of these medications were given daily as a maintenence dose, and others were utilized only during the Prodromal or Peak phases. Psychotropic medications including anti-anxiety, anti-depressant, and anti-psychotic, and sedative medications were the most effecive in treating Peak phase symptoms (Table 3). Two other drugs were reported to be effective by multiple families in the peak phase—the alpha-agonist and anti-adrenergic medication clonidine (6/15), and medical marijuana/THC (4/15), given as THC only, THC + CBD oil, or liquid via G-tube.

**Table 3:** Details of empirical medications, categorized by class and by efficacy in Peak phase

Medication class	Number of patients	Single most effective medicine for each patient during peak of episodes	Additional effective medications^a^ endorsed by parents
Non-opioid painkillers	5	Paracetamol (2)	
Opioid painkillers	5		Hydromorphone Oxycodone
Psychotropic medications and sedatives: anti-anxiety, antidepressant, anti-psychotic	10	Clonazepam (2) Lorazepam Diazepam (2) Chloral hydrate Nortriptyline	Lorazepam (3) Diazepam Chloral hydrate Clonazepam
GERD/GI medications	9	Daily enema	
Anti-epileptic, neuralgic, dystonia	11	Medical marijuana/THC (2)	Medical marijuana/THC (2) Phenobarbital
Anti-adrenergic	7	Clonidine (2)	Clonidine (4) Tizanidine
Beta blocker	2	Propranolol	Atenolol (tachycardia)
Miscellaneous	4		Atenolol (tachycardia) Ondansetron injection (severe nausea) Tetrabanazine
Non-prescription sleep aids	4		Dream (melatonin, gabapentin, chamomile extract)

^a^The medications are not mutually exclusive, and many patients were on multiple medications

In addition to medications, parents also have tried comforting their child by reducing environmental stimuli (quiet, calming environment), cuddles, rocking etc.

He can get so sensitive as we cannot look or directly talk to him and he only remains content and without cries if we keep him in a dark quiet room. **Family 1**

### NCCPC-R results

**Fig. 1:**
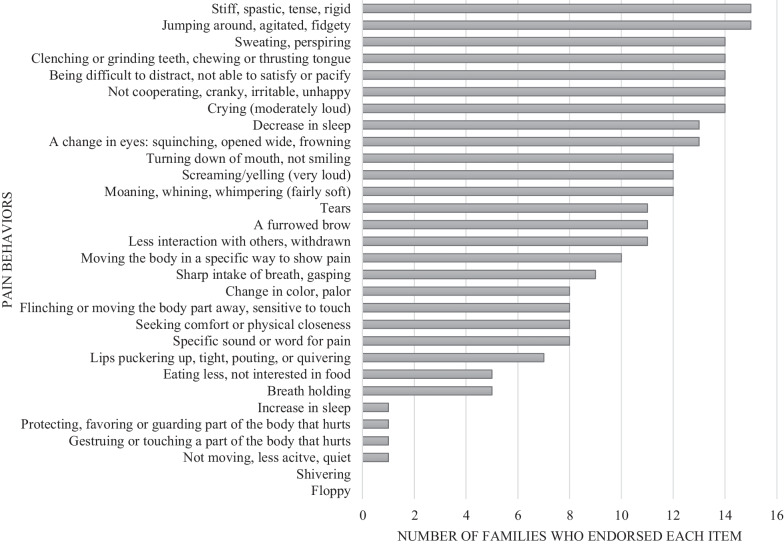
l. Parental responses to the 30 items on the NCCPC-R, illustrating the multiple and frequent indicators of pain in the affected individuals

The surveys were completed by all families (15/15). Figure 1 illustrates the frequencies for each item on the NCCPC-R. The mean total score was 51.9 ± SD 8.1 (37 to 64), and a median of 56. Remarkably, all 15 individuals had scores higher than the NCCPC-R cutoff of 7, which indicates pain. All the parents endorsed that their children were stiff/spastic/tense/rigid and jumping around/agitated/fidgety (Fig. 1).

### Impact on families

The extreme nature of the episodes, the medical impact on the child and the lack of an etiology and specific treatment is theorized to result in an impact on the families. Indeed, we found that nearly all parents report a tremendous impact on the well-being of their family, including an emotional impact (12/14), practical/logistical challenges of everyday life (13/14), and impact on extended family and/or siblings (8/14) (Table 4). Although not specifically asked through prompts, three families also described medical uncertainty and concern about the future.

**Table 4:** Representative quotes describing the impact of the irritability episodes on families

*Emotional impact on families* As a parent, it is heart-wrenching to watch your child scream in such a way that he communicates that he is being tortured. And when nothing you do helps, you get depleted, depressed, and it brings on a challenge that no parent should ever have to endure. **Family 1** The emotional toll is the worst. It is a mental torture to hear and see your child cry for so long. When he is having a good time period, you are constantly wondering (on pins and needles) when he will become upset. **Family 9** It is so sad since I remember very few happy times. We have pictures of some happy times and I barely remember anything of them due to complete exhaustion and overwhelming stress…. I have mostly stopped dreaming and frequently just exist to keep [his] needs met. It really is not mentally healthy to raise a child with this condition. **Family 6** Suffering in this way as a person can affect your relationships, your marriage, your friendships, and your parenting. You need to find a lot of mental and emotional support which can be hard to do because you feel like a broken record over and over expressing the feelings you are going through and being a voice for our son who is suffering more than any of us. You also need to lean on one's strong sense of faith to get through this kind of hardship. **Family 1**
*Practical/logical challenges caused by extreme irritability episodes* These cycles of crabbiness have ruined many aspects of our lives. We never know what version of [our son] we would wake up to in the morning which created a lot of anxiety related to whether one of us would have to stay home from work. Financially we have had to give up a lot. My husband has never worked full time since [our son] was born since we need at least one of us to have a lot of flexibility to stay home with him. **Family 6** Overall, it is so challenging to look after a child like [our son], as our brain is constantly working on something about him. It could be either about his medications or his doctor appointments or therapies or feeds or something like that. **Family 10** Extreme negative impact on [our daughter] and our family. She can scream for 24 h or more. She is unable to participate in activities she likes and we can't take her out. **Family 12**
*Impact on siblings and extended family members* It might mean that the other children go with one parent while the other parent takes care of [our son]. Or it means that care for the other kids is put on the back-burner while [he] is cared for. **Family 4** It is so frustrating to see him like that. Everyone in the family gets upset when he gets irritable and starts to scream all day for few days. **Family 10**
*Medical uncertainty and concern about the future* I'm afraid to try new medications as once [our son] had to undergo of resuscitation due to drug overdosing. When looking at all the medication he is having makes us so upset. We often think, what his future would look like if he is having a hard life when he is just 4 years. Specially when it happens continuously since birth it has been so difficult for me and my husband to plan things for the family. For the last 4 years and 4 months he has been admitted to the hospital more than 40 times. **Family 10** We felt complete and utter helplessness. We had very limited support from the medical community because of their lack in understanding of what the true problem was, let alone their disbelief in what we described. It made us feel very alone and isolated. **Family 1**

## Discussion

Individuals with NECFM caused by the recurrent *NACC1* variant c.892C>T have a unique, ubiquitous and debilitating manifestation of episodic irritability [1]. Nearly all parents describe extreme episodes of irritability, agitation and inconsolability that result in disruption of their daily activities, feeding, sleep, and require multiple medications that are typically not utilized in the management of individuals with severe neurodevelopmental disorders (outside of behavior/sleep). The episodes generally consist of three parts, including prodromal, peak, and resolving phases, and between episodes the children are described as being content, emphasizing that the irritability is cyclical and not chronic. Although most parents have not identified triggers, over time they have identified medications or environmental changes that shorten or decrease the intensity of the irritability episode.

The cause of the episodic irritability is unknown, but we considered several possibilities based on parental feedback. Parents consistently endorsed pain-related behaviors, but without an identified underlying source of the pain. While a score of seven or more on the NCCPC-R has been shown to indicate a child is in pain, the families in this study reported pain behaviors ranging from five to nine times higher than this, with a median score of 56. Parental narratives provided further details and corroborated these scores. While it is conceivable that medical problems experienced by these individuals could explain the pain behaviors, such as seizure activity and gastrointestinal problems, they did not consistently coincide with the irritability episodes making central neuropathic pain (mediated by the GI tract) and visceral hyperalgesia unlikely, and other underlying sources of nocioceptive pain were not identified. The parental description of changes suggestive of dysautonomia, such as tachycardia, sweating, and dystonic posturing raise the possibility of pain due to paroxysmal sympathetic hyperactivity (PSH). The classical occurrence of PSH is with traumatic brain injury, but in this setting the PSH episodes are usually associated with a trigger, unlike the episodes in NECFM which are cyclical and lack observable triggers [13, 14]. Moreover, we did not see brainstem lesions in our cohort with NECFM; instead they had global delayed myelination and volume loss [9, 14, 15]. We also considered other pain causes such as spasticity, dystonia, muscle spasms, and delirium, but these are also not consistently reported in parental narratives.

Due to the cyclical nature of the episodes, we considered episodic syndromes of childhood associated with migraine, including abdominal migraine, benign paroxysmal vertigo, and cyclic vomiting syndrome. These cyclical episodes may include irritability, photophobia, phonophobia, decreased oral intake, vomiting, and/or dystonic posturing, and a reduced quality of life [16, 17]. The episodes often include premonitory symptoms and a postdrome phase, similar to episodes in the NECFM cohort [18]. Interestingly, there are associations between babies who have colic, originally termed “paroxysmal fussing in infancy”, and those who later develop migraines in childhood, indicating a possible underlying central neurologic source [19, 20]. Some individuals in this study preferred being in a quiet, dark room during the peak of the episodes. However, other symptoms of migraine such as vomiting and cranial autonomic symptoms such as conjunctival injection and rhinorrhea were not evident in our cohort during the episodes and so we do not have enough evidence that migraine variants are the underlying cause of episodic irritability in NECFM [17]. Overall, although there is overwhelming endorsement of pain-related behaviors by families, corroborated by the high scores on the validated pain scale, it remains unknown whether pain is the primary underlying cause of the episodes or if there could be pain and non-pain related etiologies; additionally, we do not know the exact underlying cause, if indeed the cyclical episodes are due to pain.

Although no medication has been identified that eliminates the irritability episodes, parents reported that some medications do reduce the intensity of the symptoms in some patients. The most common group of medications reported by parents to be efficacious included psychotropics and sedatives, particularly benzodiazepines including clonazepam, diazepam, and lorazepam. This drug class potentiates gamma-aminobutyric acid (GABA) neurotransmission by binding to the GABA receptor complex and inhibits the sympathetic nervous system response, thus slowing the central nervous system. Other mood stabilizing medications that were reported by parents to be helpful include a hypnotic (chloral hydrate) and tricyclic antidepressant (nortriptyline). In addition, clonidine, also reported as effective by some families, attenuates sympathetic nervous system responses and is often used in individuals with severe neurologic impairment as a treatment for neuroirritability. Similar effectiveness was reported in a small number of individuals with Baker-Gordon syndrome [12]. Beta-blockers also seem to alleviate symptoms. Review of the literature indicates that there is significant overlap between the medications found to be helpful in PSH and in our cohort of patients with NECFM (beta-blockers, benzodiazapenes, clonidine) [21].

Medical marijuana in the form of tetrahydrocannabinol (THC) and cannabidiol (CBD) was reported by four families to offer some relief, although availability varies widely between countries. Plant compounds derived from the Cannabis sativa plant, including THC and CBD, have been successfully used for various neurological symptoms, including spasticity due to multiple sclerosis, seizures and anxiety [22–25]. THC, CBD and related compounds are also thought to modulate neurotransmission in the glutamatergic, GABAergic, and serotonergic systems [22, 25]. One study showed that a single dose of CBD reduced blood pressures in healthy subjects, suggesting that CBD may cause sympathoinhibition [26]. However, the use of CBD or THC and its modulation in the sympathetic nervous system, deserves further study.

Finally, this disorder has a profound impact on all aspects of family life. There is a need for heightened awareness in medical community. Since NECFM is a relatively newly described disorder and is ultra-rare, families have no evidence-based guidelines on effective medications and instead embark on individual and long journeys to find relief for their children. They report that over time they have learned how to better support their children and treat the symptoms through medication and environmental changes. It is unknown whether earlier effective intervention may alter the course of escalation of the irritability episodes, but identification of a prodromal period as described by parents in this study is a first step toward investigation this possibility, with further studies including objective medical data needed. Partnership with medical providers, particularly palliative care specialists, is critical for these families, and may help in assisting them to identify caregivers who can understand and help manage the episodes. Undoubtedly, more individuals with this ultra-rare disorder will be diagnosed over time, due to large scale research sequencing of individuals with undiagnosed disorders, such as in the UDN, as well as with widespread clinical exome sequencing. Indeed, the cohort of individuals with the recurrent *NACC1* variant c.892C>T has increased to over 30 that we are aware of, after the initial publication [1]. Further studies are needed to understand the pathophysiology of this disorder, in order to design targeted treatment with the goal of mitigating and ultimately preventing the devastating episodes.

There were limitations to this study. Only families of individuals with the recurrent *NACC1* variant c.892C>T were included in this study. The authors are also aware of more than 10 individuals with a variant different than the one reported here, who have not experienced these extreme irritability episodes, suggesting a variant-specific phenotype. We decided to only include individuals with the recurrent variant, so as to delineate the episodes without the confounding bias of genotype effects on the clinical course of these. The NCCPC-R was chosen as a measure to address the challenge of assessing pain in these children who are unable to communicate due to profound neurodevelopmental impairment. However, since pain is not confirmed to be the underlying cause of the behaviors, scores should be interpreted with caution. We do not have data beyond pain behaviors that substantiates or refutes other possibilities such as PSH. Further study using formal physiological measurements may provide clarity on the etiology, but are challenging to obtain, but the perspectives of neurologists that are treating these individuals may be of value in the future. Finally, information presented here is from the perspective of parents, and formal review of medical records was not performed, but could be pursued through a future study.

## Conclusions

In conclusion, individuals with NECFM caused by the recurrent c.892C>T variant in *NACC1* have episodes of extreme irritability that are incapacitating and require major medical interventions with multiple medications, causing major hardship for the affected child and family members. All parents endorse significant pain behaviors during these episodes, and the underlying cause remains unknown. It is encouraging to note that the episodes stabilize, improve or are managed more effectively as the child ages. Support and understanding from the medical community is needed, as well as further research to better understand the pathophysiology of the episodes and how to more effectively treat these, for the betterment of the children and their families.

## Methods

Participants included parents of individuals from and outside the UDN, confirmed to have the recurrent *NACC1* variant previously reported by Schoch et al. (NM_052876.3:c.892C>T; p.(Arg298Trp)) [1]. All participants provided written informed consent to participate (Duke IRB-approved Protocol Pro00100610). The participants were recruited from the NACC1 family support group page on Facebook. The study was retrospective in nature, with parental recollections of the episodes informing their narratives and answers to the pain survey.

### Measures

Parental narratives of the episodes and a pain survey were selected to ascertain the clinical features of the episodes. Subsequently specific questions were added to describe physiological changes during episodes and clarify medication efficacy and seizure management. Responses were collected via REDCap (Research Electronic Data Capture) [27].

### Narratives

Open-ended prompts were designed, informed by prior anecdotal information from parents, to better characterize the irritability episodes (See Parental Narrative Prompts in Additional file 1). The five prompts were to describe: (1) the episodes in terms of age of onset, frequency and duration, course over time, signs of an upcoming episode, behavioral changes and methods used by the family to track these for accurate recollection; (2) symptomatic associations, triggers, and/or environmental factors (external to patient) precipitating or preceding an episode; (3) changes in eating/appetite or bowel/bladder emptying; (4) interventions attempted and the outcomes of each; and (5) impact on the child’s and family’s quality of life. Parents were asked to type their responses in the REDCap database.

### Pain survey

The Non-communicating Children’s Pain Checklist-Revised (NCCPC-R) is a pain assessment tool designed for children unable to speak because of cognitive impairments [28]. It includes 30 behaviors across seven categories, including Vocal, Social, Facial, Activity, Body and Limbs, Physiological, and Eating/Sleeping. The observer is asked to rate the child’s behavior on a Likert scale from 0 (Not at all) to 3 (Very often). The behavior scores are summed for a Total Score, and a Total Score of 7 or more indicates a child has pain [28]. Parents were asked to complete the questionnaire considering behaviors during a typical episode of irritability. If an item did not apply to the child, the parents were instructed to mark “not applicable” for that item.

Quantitative data collected from the NCCPC-Rwere analyzed with SPSS using descriptive methods (IBM SPSS Statistics, version 26). Qualitative responses based on the parental narratives were analyzed using directed content analysis with ATLAS.ti (version 7.8, http://atlasti.com) [29–31]. This qualitative analytic approach enables coding and subsequent themes to be developed directly from the data without a prior theoretical model. Narratives were read and reread by KS and AMR, and iterative process was used to develop codes with new codes added as needed. KS and AMR coded independently and then jointly until agreement was reached and findings reviewed with VS. Once coded the data were then sorted, tabulated, and summarized. This approach was used as it provided rich descriptive of the parental perspectives of the episodes. The attempted management of the episodes, including medications used, were reviewed by QT, KS, and VS.

### Supplementary Information

**Additional file 1.** Parental Narrative Prompts.

## Data Availability

The data that support the findings of this study are not openly available due to reasons of sensitivity and are available from the corresponding author upon reasonable request. Data are located in controlled access data storage at Duke University School of Medicine.
