# Supplementary material for: Parental perspectives of episodic irritability in an ultra-rare genetic disorder associated with NACC1
Source: Orphanet J Rare Dis. 2023 Sep 4;18:269. doi: 10.1186/s13023-023-02891-3 (PMC10476425; doi:10.1186/s13023-023-02891-3)
Supplement: Supplementary file 1 — Additional file 1. Parental Narrative Prompts. [file 13023_2023_2891_MOESM1_ESM.docx]

**Supplemental Information**

Parental Narrative Prompts

Severe and episodic irritability or inconsolability is a common symptom in individuals with NACC1-related neurodevelopmental disorder, yet poorly understood by the medical community. Please provide a response to the following prompts in your own words. Please provide as much detail as possible.

You may complete your narrative on this page, or if you prefer you may upload a word or text file at the bottom of this page.

Detailed description of a 'typical' episode or cycle:

- Timing of the episodes, including age of onset, frequency and duration
- Course over time (getting worse, better or the same and any other change in nature of episode)
- Signs or clinical features (if any) of an upcoming episode
- Behaviors that emerge or are more severe during an episode
- Communication differences during an episode
- Family methods for tracking information about cycles, if any

2) Associations, triggers, and/or environmental factors you may have noticed preceding an episode, such as behavioral or movement changes, or personality or temperament changes, if any. Please describe if these associations have changed over time.

3) Changes in eating/appetite and/or frequency of bowel and/or bladder emptying during these episodes, if any.

4) Interventions attempted to reduce impact of episodes, including medications/supplements, environmental adjustments, and/or medical therapies, and their outcomes. Please describe whether the efficacy of these has changed over time.

5) Impact on the child's and family's well-being and/or quality of life during these episodes.

Have any of the following symptoms/changes been documented during the episodes or cycles? If readily available please provide details.

- Increase or decrease in heart rate
- Increase or decrease in blood pressure
- Increase or decrease in temperature
- Change in skin color
- Increase or decrease in sweating
- Increase or decrease in muscle tone
- Posturing of extremities or trunk
- Increase or decrease in respiratory rate (breathing)

Please rank the top 1-2 medications that have helped with easing symptoms during an episode or cycle.

If there are no medications that you have found helpful, please write “None.”

Has your child had seizures (at any time, not only during an episode or cycle)?

Are your child's seizures well-controlled at this time?

When was your child's last seizure?

Have you or any physicians suspected seizure activity before/during the episodes or cycles?

Has an EEG been performed during an episode or cycle?

Did the EEG show seizure activity?

How would you characterize your child's episodes or cycles now (considering frequency and severity) compared to when the child was < 3 years old?
